# Organophosphorus Poisoning among Acute Poisoning Cases Presenting to the Emergency Department of a Secondary Care Centre: A Descriptive Cross-sectional Study

**DOI:** 10.31729/jnma.7446

**Published:** 2022-05-31

**Authors:** Subhash Pandey, Nitesh Shrestha

**Affiliations:** 1 Department of Emergency Medicine, Bardiya Hospital, Gulariya, Bardiya, Nepal; 2 Department of General Medicine, Kaushalya Memorial Hospital, Kohalpur, Banke, Nepal

**Keywords:** *acetaminophen*, *emergency departments*, *organophosphorus poisoning*, *prevalence*

## Abstract

**Introduction:**

Organophosphorus is an easily available compound, especially in agriculture and farming related areas. This study evaluated organophosphorus poisoning among the population in those high-risk areas. The main objective of this study is to find out the prevalence of organophosphorus poisoning among acute poisoning cases presenting to the Emergency Department of a secondary care centre.

**Methods:**

A descriptive cross-sectional study was conducted among 427 patients presenting to the Emergency Department in a secondary care centre from 17^th^ July, 2018 to 14^th^ January, 2022. Ethical clearance was taken from the Institutional Review Committee (Reference number: 01/2075-76). All the patients presenting to the Emergency Department were included and the patients without consent, patients with trauma, accident, severe illness and other emergency conditions were excluded. A convenience sampling was done. Data were collected and entered in Microsoft Excel version 2007 and analyzed using Statistical Package for the Social Science version 25.0. Point estimate at 95% Confidence Interval was calculated along with frequency and percentage for binary data.

**Results:**

Out of 427 patients, 203 (47.54%) (42.80-52.28 at 95% Confidence Interval) had organophosphorus poisoning. It was most commonly seen in the age group 16-30 years among 103 (50.74%).

**Conclusions:**

The prevalence of organophosphorus poisoning in our study was similar when compared to other studies conducted in similar settings. Most of the organophosphorus poisoning cases were intentional and suicidal which is similar to other studies.

## INTRODUCTION

Poisoning is the second most common method of suicide in Nepal,^1^ and over 24% of these suicides in Nepal are due to ingestion of highly concentrated agricultural pesticides^2^ mostly Organophosphorus (OP).^3^ OP are commonly used for agriculture and domestic pest control which may lead to poisoning by unsafe use, accidental drinking, or suicide attempts, especially in farming areas.^4,5^ Bardiya is in the midwestern part of Nepal where most people are engaged in agriculture, so OP are easily available, and it is the preferred means for poisoning.

OP poisoning patients are increasing almost every day. On-time diagnosis of types of poison ingestion and treatment with the suitable antidote and symptomatic treatment can minimize the severity, morbidity, and mortality of the patients.

This study aims to find out the prevalence of organophosphorus poisoning among acute poisoning cases presenting to the Emergency Department of a secondary care centre.

## METHODS

A descriptive cross-sectional study was conducted in the Emergency Department of Bardiya Hospital, a secondary care centre from 17^th^ July, 2018 to 14^th^ January, 2022. Ethical clearance was taken from the Institutional Review Committee of Bardiya Hospital (Reference number: 01/2075-76). Patients from age <16 to age >60 years were included and divided into 5 groups, the age groups from <16, 16-30, 31-45, 46-60, and >60. Acute poisoning by organophosphorus was diagnosed by acute exposure of fewer than 24 hours with the clinical features of increased saliva and tear production, diarrhoea, nausea, vomiting, small pupils, sweating, muscle tremors, and confusion. The inclusion criteria in this study were all the patients present in the ED with acute poisoning. The exclusion criteria include those patients or their relatives who were unwilling to give consent, patients with trauma, accident, severe illness and other diseases that lead patients to ED. Convenience sampling was done and the sample size was calculated using the following formula:

n = (Z^2^ × p × q) / e^2^

  = (1.96^2^ × 0.046 × 0.954) / 0.02^2^

  = 422

Where,

n = minimum required sample size,
Z = 1.96 at 95% Confidence Interval (CI)
p = prevalence of organophosphorus poisoning among acute poisoning, 4.6%^1^
q = 1-p
e = margin of error, 2%

The minimum required sample size was 422. However, a total of 427 sample size was taken for the study and data regarding the age, sex, the reason for taking poison, residence, occupation, and types of disposal of poisoned patients were recorded in the proforma.

Data were entered in the Microsoft Excel version 2007 and analyzed in the Statistical Package for the Social Sciences (SPSS) software version 25.0. Point estimate at 95% Confidence Interval was calculated along with frequency and percentage for binary data.

## RESULTS

**Figure 1:**
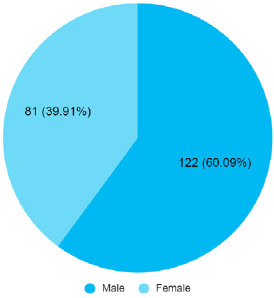
Distribution of organophosphorus poisoning cases by sex (n= 203).

Out of 427 patients, 203 (47.54%) (42.80-52.28 at 95% Confidence Interval) had OP poisoning among which 122 (60.09%) were females and 81 (39.91%) were males with a ratio of 1.5:1 (Figure 1).

Organophosphorus poisoning was most commonly seen in the age group 16-30 years among 103 (50.74%) followed by patients between 31 to 45 years of age in 49 (24.14%). Forty-three (21.18%) cases were accidental, and the patients were most commonly from the semi-urban setting in 92 (45.32%). Seventy-three (35.96) patients were students. One-hundred twenty (59.11) patients were referred to higher centres for management (Table 1).

**Table 1:** Demographic details of the patients with organophosphorus poisoning (n = 203).

Categories	n (%)
**Age of patient**	
<16	24 (11.82)
16 to 30	103 (50.74)
31 to 45	49 (24.14)
46 to 60	23 (11.33)
>60	4 (1.97)
**Reason for taking poison**	
Accidental	43 (21.18)
Suicidal	115 (56.65)
Not known	45 (22.17)
**Residence**	
Urban	69 (33.99)
Semi-urban	92 (45.32)
Rural	42 (20.69)
**Occupation**	
Student	73 (35.96)
Housewife	44 (21.67)
Farmer	29 (14.29)
Service holders	31 (15.27)
others	26 (12.81)
**Outcome of the patients**	
Admitted and discharged from ED	65 (32.02)
Refer	120 (59.11)
Expired	18 (8.87)
**Total**	203 (100)

## DISCUSSION

Organophosphorus poisoning is one of the most frequent causes that can lead patients to visit ED. In our study, 47.54% had OP poisoning among all poisoning presenting to the ED. One study conducted in China reported that OP poisoning accounts for 17.2% of pesticide category poisoning,^6^ 43.5% in the eastern part of Nepal^7^ and 32.8% in India.^8^ This study showed that the incidence of poisoning in women was slightly higher than in men, with the female to male ratio of 1.5:1 which is consistent with other studies with similar settings.^6,7,9^

In this study, we found that in the age group 16-30, the ingestion of OP poison was the most common, and in the age group >60, the ingestion of poison was least, which was 50.74% and 1.97% respectively. One study found that the most vulnerable to poisoning were those aged 20-29 years.^6^ While in another study conducted in the eastern part of Nepal, 56.4% of the patients with poisoning presented within the age group of 15-30.^7^ The findings of our study were consistent with those studies. The poisoning is more common among the age group less than 30 years, reflecting their exposure to stress, possibly due to failure or frustration in their study, personal relationships, exams, or jobs.^10^ In our study, OP poison ingestion was higher among students (35.96%), while 21.67% were housewives, 14.29% were farmers, 15.27% were service holders, and 12.81% were in other occupations which is consistent with the study conducted in the tertiary care centre of Nepal.^11^

In our study, most patients' residences were in urban areas (33.99%), followed by semi-urban, 45.32%, and rural, 20.69% which is less than other similar studies.^6,12^ Asking the patients about the reasons for taking poison and most of the patients were suicidal 56.65%, 21.18% were accidental, and 22.17% were not known. A study reported that 94% of poisoning was due to suicidal purposes.^8^ In another study 88% of pesticide poisonings were used for suicide attempts which is more when compared to our study.^6^

In our study, 32.02% were admitted and discharged from the ED of our hospital and 59.11% were referred. This finding is similar to other studies.^6^ In our study referred rate is higher due to the unavailability of Intensive Care Units (ICU) and limited resources. The mortality by OP poisoning in our study was 8.87% which was comparable to the mortality by poisoning cases observed in other studies.^6,9^

The sample is specifically limited to the patients attending ED of Bardiya Hospital, mid-western Nepal. Findings are limited because they are based on a limited population at one site. The other serious limitation is common to all studies that use the information provided by patients and their relatives. Also, a higher sample size could increase the generalizability of the findings from the study.

## CONCLUSIONS

The prevalence of our study was similar when compared to other studies in similar settings. The present data gives an insight into the prevalence of OP poisoning and could represent a trend in mid-western Nepal. Most of the OP poisoning cases were intentional and suicidal in nature and more common among women. On-time diagnosis of OP poison ingestion and treatment with the antidote and symptomatic treatment could minimize the severity, morbidity, and mortality of the patients.
